# Insertion of Fluorescent Proteins Near the Plug Domain of MotB Generates Functional Stator Complexes

**DOI:** 10.1002/mbo3.70056

**Published:** 2025-09-15

**Authors:** Jyoti P. Gurung, Pietro Ridone, Anaïs Biquet‐Bisquert, Gary Bryant, Francesco Pedaci, Ashley L. Nord, Matthew A. B. Baker

**Affiliations:** ^1^ School of Biotechnology and Biomolecular Science UNSW Sydney Sydney Australia; ^2^ Centre de Biochimie Structurale (CBS), INSERM, CNRS Université de Montpellier Montpellier France; ^3^ Department of Physics, School of Science RMIT University Melbourne Australia

**Keywords:** bacterial flagellar motor, fluorescence, LOV domain, MotB, stator complex

## Abstract

Many bacteria swim by the rotation of the bacterial flagellar motor (BFM). The BFM is powered by proton translocation across the inner membrane through the heteroheptameric MotA_5_MotB_2_ protein complex. Two periplasmic domains of MotB are critical in activating BFM rotation: (1) the peptidoglycan (PG) binding domain that anchors MotB in the PG layer and (2) the plug domain that modulates the proton flow. Existing cytoplasmic fluorescent probes have been shown to negatively affect motor rotation and switching. Here, we inserted a fluorescent probe in the periplasm near the plug of MotB to circumvent issues with cytoplasmic probes and for possible use in observing the mechanism of plug‐based regulation of proton flow. We inserted green fluorescent protein and improved light‐oxygen‐voltage (LOV), a fluorescent version of the LOV domain, in four periplasmic locations in MotB. Insertions near the plug retained motility but showed limited fluorescence for both fluorophores. Additional short, flexible glycine–serine linkers improved motility but did not improve brightness. Further optimization is necessary to improve the fluorescence of these periplasmic probes.

## Graphical abstract

We inserted small light‐interacting domains at various locations in the stator complex to see what sites were amenable for labeling and how these affected flagellar rotation and switching. We observed a site near the plug domain to be the best labeling site and were able to label this site with a small light‐oxygen‐voltage domain that was weakly fluorescent in the periplasm.

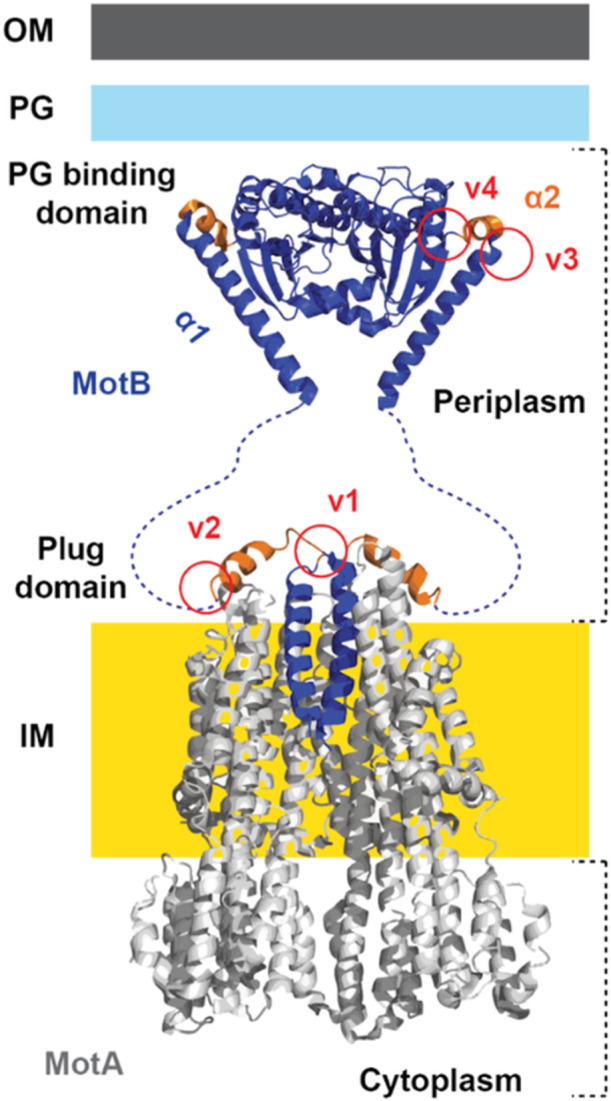

## 1 Introduction

**Figure 1:**
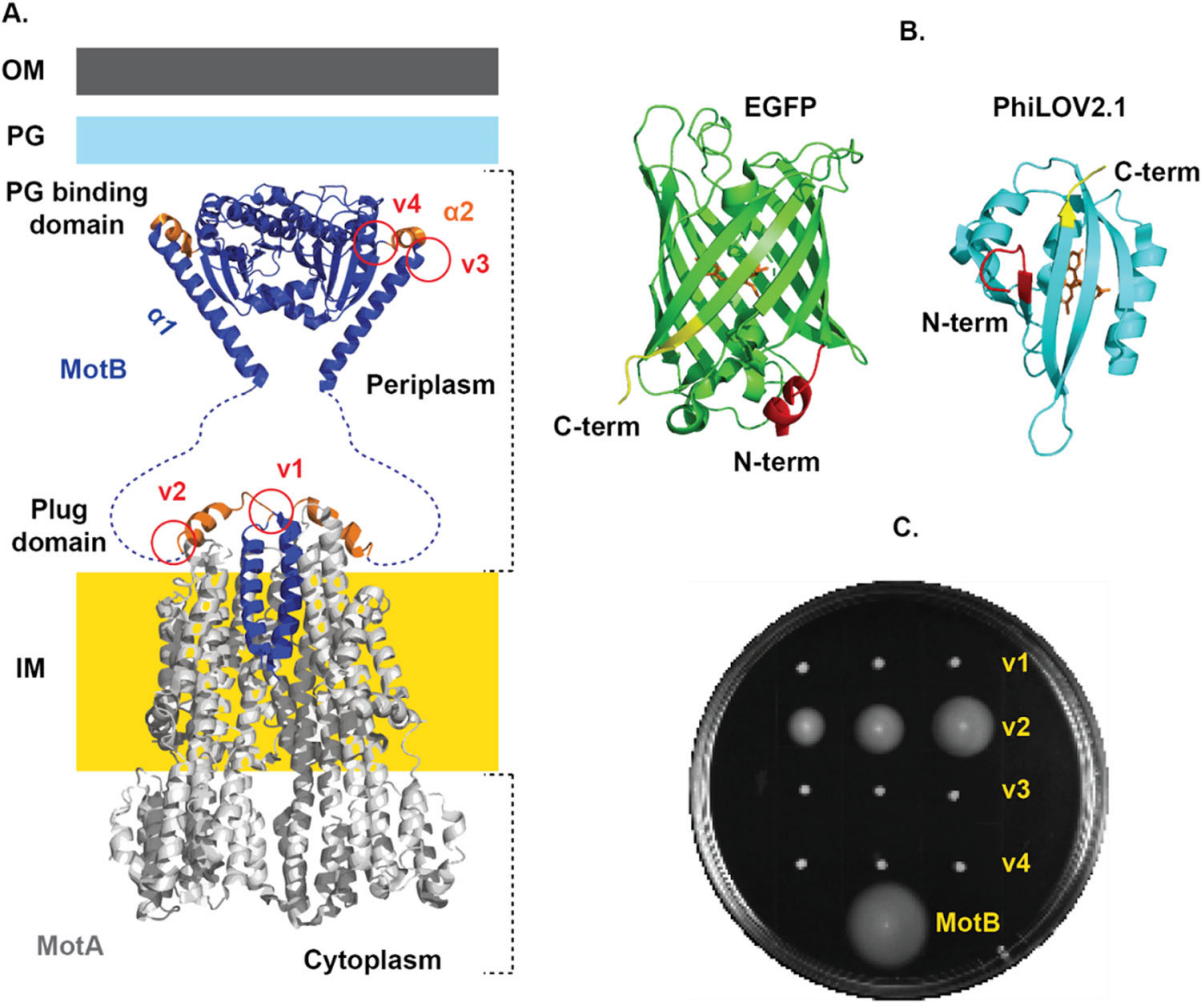
Green fluorescent proteins (GFP and iLOV) insertion sites in MotB and its impact on bacterial motility. (A) Structure of MotA_5_MotB_2_ stator complex in the bacterial cell membrane. Cryo‐EM structure of the stator complex containing MotA (gray‐colored) and MotB (blue‐colored) with PG binding domain from *Salmonella enterica* (PDBID: 2zvy), and plug and transmembrane (TM) domain from *Campylobacter jejuni* (PDBID: 6ykm). Location of the LOV domain in MotB (denoted by the red solid circle). v1 and v2 represented the insertion of the LOV domain before and after the plug domain (orange‐colored), respectively. v3 and v4 represented the insertion of the LOV domain before and after the short α2‐helix of the PG binding domain (orange‐colored), respectively. (B) Crystal structure of enhanced green fluorescent protein (EGFP) with N‐terminal (red), barrel‐shaped core (green), tripeptide sequence Threonine65–Tyrosine66–Glycine67 as chromophore (orange), and C‐terminal (yellow) derived from *Aequorea victoria* (PDBID: 2Y0G). Crystal structure of fluorescent LOV domain (iLOV) with N‐terminal (red), middle core (light blue), FMN as chromophore (orange), and C‐terminal (yellow)derived from *Arabidopsis thaliana* (PDBID: 4EEU). (C) Image of swim plates, containing semisolid media (0.3% agar) with 1.33 mM l‐arabinose after 24 h of incubation at 30°C. Bacterial strains: MotB tagged with LOV (v1, v2, v3, and v4), and wild‐type *Escherichia coli* motile strain as a positive control (MotA_5_MotB_2_ expressing *E. coli* strain). Three colonies for each bacterial strain were inoculated in a swim plate (except for the wild‐type *E. coli* strain). Cryo‐EM, cryo‐electron microscopy; FMN, flavin mononucleotide; iLOV, improved light‐oxygen‐voltage; IM, inner membrane; LOV, light‐oxygen‐voltage; OM, outer membrane; PDBID, Protein Data Bank Identity; PG, peptidoglycan.

The bacterial flagellar motor (BFM) is a molecular machine responsible for motility in many bacteria, such as *Escherichia coli*, driving the rotation of flagella to propel cells through their environment. The rotation of the BFM is powered by the translocation of ions, including H⁺ and Na⁺, through transmembrane protein complexes known as stator units. These stator complexes are classified as MotAB and PomAB, depending on the type of ion, H⁺ or Na⁺, respectively, that is translocated. MotA_5_MotB_2_ stator complex specifically utilizes proton flow to generate torque through its interactions with the rotor, facilitating the rotation of the motor (Deme et al. 2020; Santiveri et al. 2020). The N‐terminal of the MotB monomer has a small cytoplasmic tail, then a single transmembrane helix in the inner membrane followed by a short helix (the plug domain), and an unstructured linker, which is finally connected to a peptidoglycan (PG) binding C‐terminal domain (Figure 1). The plug domain has been demonstrated to play a pivotal role in the activation of the rotor in the BFM by modulating ion flux (Homma et al. 2021). It is hypothesized that upon MotB binding to the PG layer, the helical structure of the “plug domain” releases from the periplasmic surface of MotA, exposing the ion pore and facilitating the route for H^+^ ion translocation (Hu et al. 2023). The PG binding domain is critical in assembling the stator complex around the BFM rotor by anchoring it to the PG layer. The stator is dynamic, and the off‐rate of stator binding is modulated in response to increased load (Lele et al. 2013; Nord et al. 2017).

Fluorescent protein labeling can offer insight into single‐molecule binding events of the stator complex. To date, standard fluorescent proteins, such as green fluorescent protein (GFP), which are relatively large (~27 kDa), have been inserted in the N‐terminal of MotB (i.e., cytoplasmic labels) (Leake et al. 2006; Tipping et al. 2013) and have been shown to impact the rotational speed and switching of the flagellar motor (Heo et al. 2017). In our work, we selected a fluorescent version of the light‐oxygen‐voltage (LOV) domain, that is, improved LOV (iLOV) protein as a small periplasmic fluorescent probe for MotB (Christie et al. 2012). The iLOV fluorescent protein has an advantage over standard fluorescent proteins such as GFP due to its size (~11 kDa, 115 amino acids long), its oxygen‐independence (it can fluoresce in anaerobic conditions) and functionality under a broad range of pH (4–11) (Drepper et al. 2007; Swartz et al. 2001).

The precursor of iLOV, that is, LOV, is a photo‐responsive domain containing flavin mononucleotide as a cofactor or chromophore for photo‐sensitive activity. LOV domains are present in many kingdoms of life, predominantly in bacteria and plants with diverse functionalities (Crosson et al. 2003). The LOV domain is composed of three motifs: an N‐terminal short helix (A′‐α‐helix), a middle LOV core (β‐sheets and α‐helix) and a C‐terminal long helix (J‐α‐helix). Flavin mononucleotide is bound to the LOV core in a dark state during which the J‐α‐helix is sequestered into the LOV core. Upon blue light exposure, the helical structure of J‐α‐helix unwinds and causes its release from the LOV core to a disordered or distended form. This conformational change of the J‐α‐helix upon light exposure can be used as an optogenetic tool, for example, the J‐α‐helix of the LOV domain was used to regulate the opening and closing of potassium channels for neuron function (Jerng et al. 2021).

Here, we inserted a photoactivable LOV domain and a fluorescent LOV domain (iLOV) at two sites near the plug domain and two sites near the PG binding domain. First, we tested the effect of blue light illumination on bacterial motility when the photoactivable LOV domain was inserted into MotB. We hypothesized that the conformational change in the LOV domain, induced by blue light, would affect motility in a measurable manner. We then tested the motility and fluorescence of fluorescent iLOV that was inserted into MotB to develop it as a potential periplasmic fluorescent probe. We hypothesized that the motility of cells powered by an iLOV‐tagged MotB stator would be comparable to wild‐type, with minimal disruption in motility compared with the GFP counterpart. Finally, to improve the weak fluorescence of iLOV compared with GFP (Mukherjee et al. 2013), we inserted short and flexible linkers in between fluorescent iLOV and MotB periplasmic domains to determine if linkers could rescue fluorescence while retaining bacterial motility.

## 2 Results

### 2.1 The LOV Domain Inserted After the Plug Domain of MotB in *E. coli* Does Not Disrupt Bacterial Motility

Four sites were selected in the periplasmic region of MotB for the insertion of photoactivable LOV domain (Figure 1A). Two were near the plug domain; one before the α‐helical plug at the 50th amino acid of MotB (henceforth known as “v1”), and one after the plug domain at the 64th amino acid of MotB (henceforth known as “v2”). The two remaining sites were before the PG binding domain at the 128th amino acid of MotB (henceforth “v3”) and after the PG binding domain at the 187th amino acid of MotB (henceforth “v4”). As a result, four periplasmic variants (v1, v2, v3, and v4), inducible by the addition of 1.33 mM l‐arabinose (see Section 5 for detailed information), were constructed, transformed into MotB knockout strains (RP3087), and the effect on bacterial motility was tested (Figure 1C). There was no impact on cell growth for all the bacterial strains we prepared (Supporting Information Figure 1). When these strains were tested on semisolid media (swim plates containing 0.3% agar and 1.33 mM l‐arabinose), periplasmic variant (v2, i.e., LOV inserted after the plug domain) spread measurably after 24 h of incubation at 30°C, whereas v1, v3, and v4 did not (Figure 1C). Furthermore, motility in swim plates exhibited by periplasmic variant (v2) and other nonmotile variants was verified in liquid media by measuring the swimming velocity using differential dynamic microscopy (DDM) (Supporting Information Table 1 and Supporting Information Figure 2). When illuminated by blue light (465 nm, 150 lx) for 24 h at 30°C, no notable differences in swim diameter on swim plates were observed for all variants compared with dark conditions (Supporting Information Figure 2). After 48 h, v3 and v4 regained motility (Supporting Information Figures 3 and 4); however, this was confirmed to be due to restored fully functional MotB from the reversion of a stop codon in the ∆motB strain (RP3087) (Supporting Information Figure 5).

### 2.2 MotB Tagged With iLOV in the Cytoplasmic (N‐Term) and Periplasmic Variants (v2) Spread Further on a Swim Plate Than the GFP Counterpart

**Figure 2:**
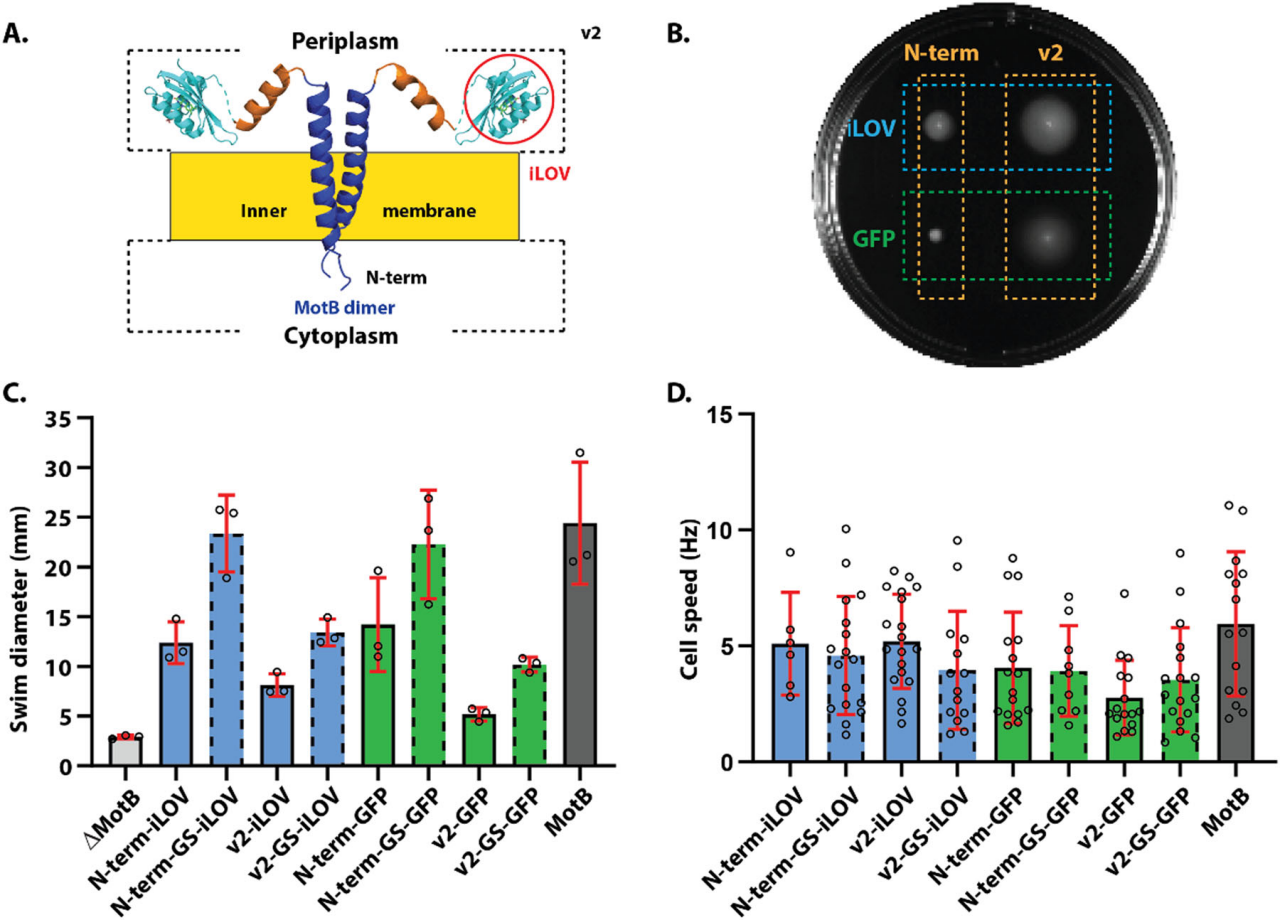
Motility assay for bacterial strains with fluorescently tagged MotB. (A) Schematic diagram of iLOV (denoted by red circle) inserted in the periplasmic domain of MotB at the v2 site. (B) Image of a swim plate (0.3% agar) showing bacterial strains after incubating for 24 h at 30°C. (C) Bar graph of swim ring diameter (mean ± standard deviation, triplicate data) measured for the bacterial strains tested. Dotted lines indicate strains with short glycine‐serine (GS) linkers. (D) Bar graph of rotational speed (mean ± standard deviation) of tethered cells for each strain. The number of cells (*n*) averaged to determine the mean rotational speed is listed in Supporting Information Table 2. The bacterial strains tested include MotB (wild‐type motile, used as “positive control” in C), ∆motB (nonmotile motB‐deleted strain, used as “negative control” in C), and sticky‐filament ∆motAB (nonmotile motA and motB deleted strain, where motA is expressed via plasmid and motB or labeled variant is expressed on a separate plasmid, with a sticky filament for tethered cell assay). The constructs for motB expressed from plasmid are: N‐term‐iLOV (MotB with iLOV tag at the N‐terminus), N‐term‐GS‐iLOV (MotB with iLOV tag at the N‐terminus with GS linker), v2‐iLOV (MotB with iLOV tag after the plug domain), v2‐GS‐iLOV (MotB with iLOV tag after the plug domain with GS linker), N‐term‐GFP (MotB with GFP tag at the N‐terminus), N‐term‐GS‐GFP (MotB with GFP tag at the N‐terminus with GS linker), v2‐GFP (MotB with GFP tag after the plug domain), v2‐GS‐GFP (MotB with GFP tag after the plug domain with GS linker), and wild‐type MotB. GFP, green fluorescent protein; iLOV, improved light‐oxygen‐voltage.

As blue light showed no effect on motility when the photo‐switchable LOV domain was inserted, we chose the periplasmic variant (v2, i.e., insertion site after the plug domain) for further development as a periplasmic fluorescent sensor since it had swimming behavior close to wild‐type. We replaced the LOV domain in the v2 variant with PhiLOV2.9 protein, an improved photostable version of the fluorescent iLOV domain (Christie et al. 2012). To assess the impact of iLOV and GFP insertions on bacterial motility, and their fluorescence intensity and utility, we inserted these fluorescent proteins at the cytoplasmic end (N‐terminal) and periplasmic site (v2) (Figure 2A).

In the periplasmic variant (v2), iLOV‐tagged MotB showed a swimming diameter of 8.1 ± 1.1 mm that was greater than its GFP counterpart (5.2 ± 0.7 mm), but smaller than wild‐type (24.4 ± 6.1 mm) (Figure 2B,C). However, the cytoplasmic variant (N‐term) showed greater motility than the periplasmic variant (v2). The introduction of glycine–serine (GS) linkers further improved bacterial motility in all variants (Figure 2C). For the cytoplasmic variant (N‐term), swim diameter of GFP‐tagged MotB increased from 14.2 ± 4.7 to 22.3 ± 5.5 mm, whereas swim diameter of iLOV‐tagged MotB increased from 12.4 ± 2.1 to 23.4 ± 3.9 mm (Figure 2C). Similarly, for the periplasmic variant (v2), a linker increased the swim diameter of GFP‐tagged MotB from 5.2 ± 0.7 to 10.2 ± 0.7 mm, whereas for the iLOV‐tagged MotB, a linker increased the swim diameter from 8.1 ± 1.1 to 13.4 ± 1.3 mm (Figure 2C). We did not observe any statistically significant difference (with *p* > 0.05) in the average rotational speed between bacterial strains with and without GS linker using a tethered cell assay (Figure 2D).

### 2.3 Rotational Comparison of iLOV‐Tagged MotB Stators With GFP‐Tagged MotB Stators

**Figure 3:**
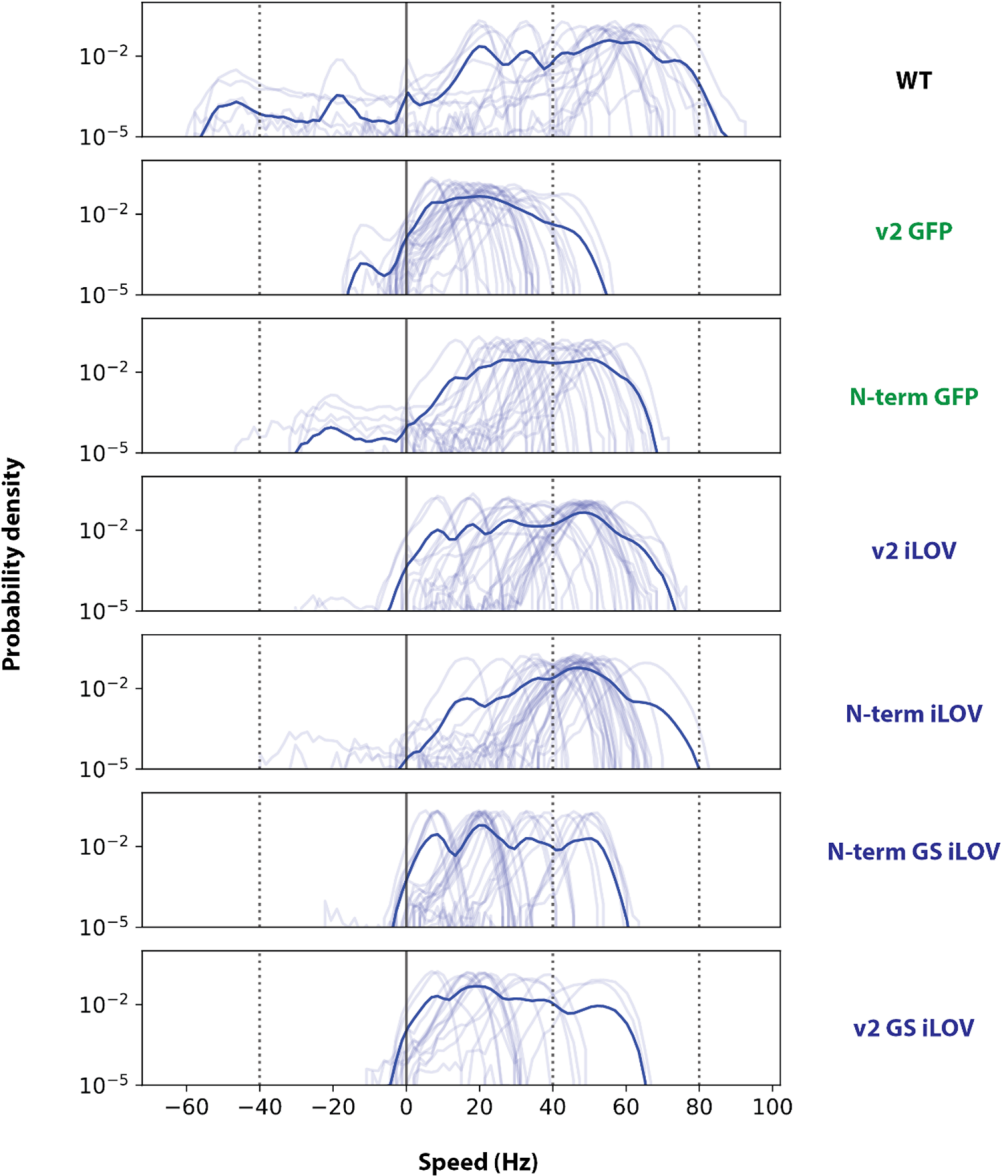
Probability density of CCW‐biased motor speeds of iLOV and GFP‐tagged MotB in N‐terminal and v2 position, measured using a bead assay. Positive and negative values of speed represent motors rotating in CCW and CW direction, respectively. Light blue lines indicate single motor speed probability densities, and thick solid blue lines indicate the mean probability density. Number of motors measured (Supporting Information Figure 6): *n* = 23 for WT, 29 for v2 GFP, 27 for N‐terminal GFP, 34 for v2‐iLOV, 34 for N‐terminal iLOV, 30 for N‐terminal iLOV GS, and 17 for v2 iLOV GS. Here, “GS” indicates the linker with two amino acids of glycine–serine. CCW, counterclockwise; CW, clockwise; GFP, green fluorescent protein; iLOV, improved light‐oxygen‐voltage; WT, wild‐type.

To assess the rotational speed and switching of the motor powered by our MotB variants, we attached 1.1 µm polystyrene beads onto truncated and hydrophobic (FliC_sticky_) bacterial filaments (Figure 3) and tracked their rotation. In the cytoplasmic variant (N‐term), we observed that the direct fusion of GFP in the N‐terminal of MotB reduced the motor speed to 37.4 ± 3.9 Hz (s.e.m) compared with the wild‐type (48.2 ± 7.6 Hz), which agreed with previous measurements (Heo et al. 2017). Direct fusion of iLOV in the N‐terminal of MotB showed motor speed of 44.7 ± 3.8 Hz, less than wild‐type but within error. Inserting a short and flexible GS linker in between iLOV and the N‐terminal of MotB decreased the motor speed to 27.3 ± 2.4 Hz. In the periplasmic variant (v2), the motor speed of iLOV‐tagged MotB was 41.0 ± 4.2 Hz, higher than the GFP counterpart (19.4 ± 4.6 Hz). As per the cytoplasmic variant, insertion of a GS linker between iLOV and the v2 site decreased the speed to 25.0 ± 3.7 Hz. For all the constructs, the maximum speeds both counterclockwise and clockwise were lower than WT rotational speed (Figure 3), and each had at least one cell with > 5 stators, indicative of high expression (Supporting Information Figures 6 and 7). Compared with wild‐type, both cytoplasmic (N‐term) and periplasmic variants (v2) showed reduced switching frequency, as seen previously (Heo et al. 2017) (Supporting Information Figure 8). The switching frequency of iLOV‐tagged MotB in the periplasmic variant (v2) was reduced in comparison with the GFP counterpart. Insertion of GS linkers in iLOV variants increased the switching frequency, most notably in the periplasmic variant (v2), but did not restore switching to WT levels (Supporting Information Figure 8).

### 2.4 Both GFP and iLOV Show Limited Fluorescence in the Periplasm Compared With the Cytoplasm

**Figure 4:**
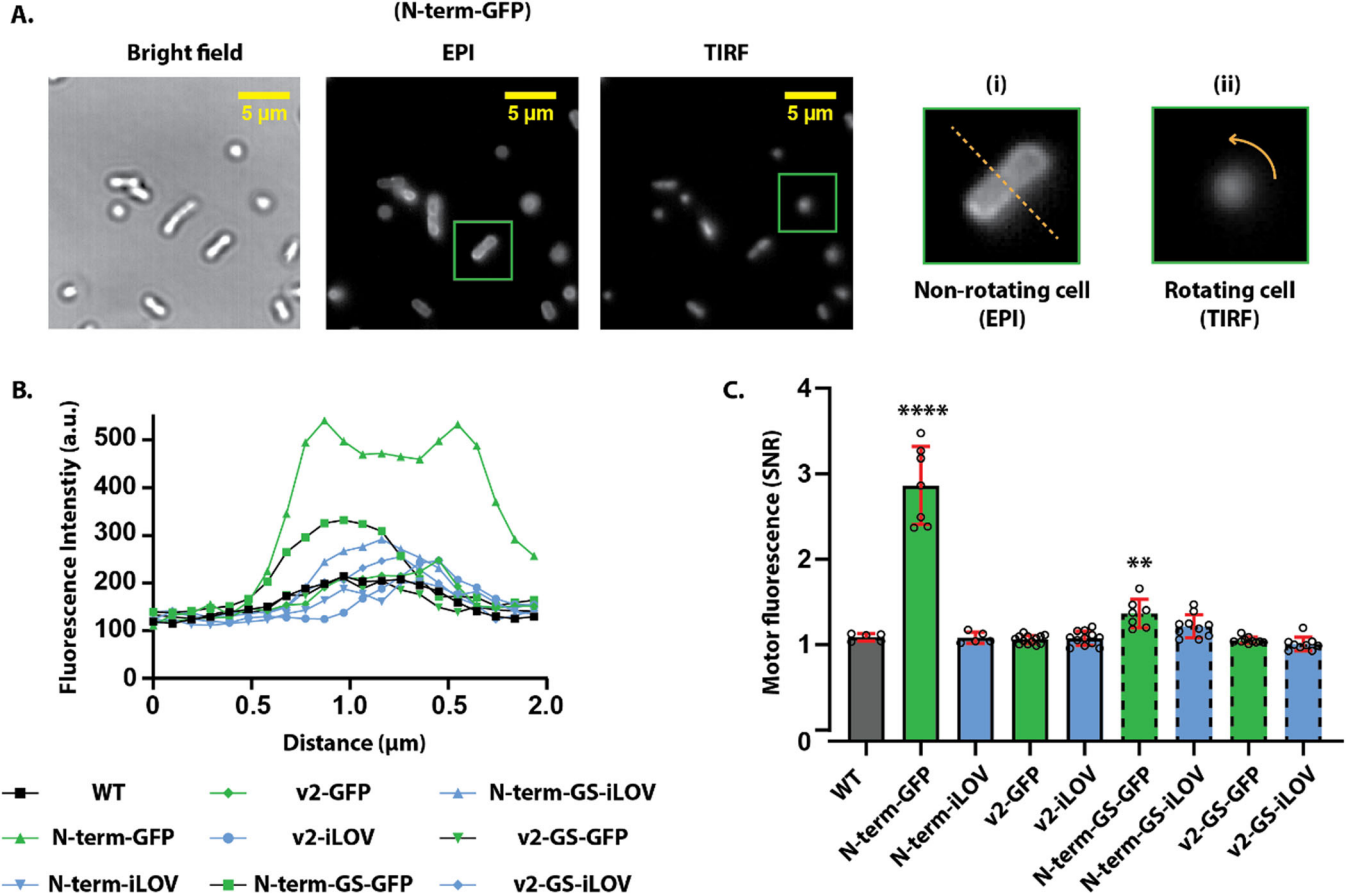
Quantification of fluorescence in GFP and iLOV‐tagged MotB constructs. (A) Representative images of bacterial cells expressing GFP‐tagged at the N‐terminal of MotB (N‐term‐GFP), the standard fluorescent MotB used in studying stator dynamics (Leake et al. 2006). Bright‐field (left), epifluorescence (EPI, middle), and total internal reflection fluorescence (TIRF) (right) images show fluorescence localization. Green boxes indicate zoomed inset regions used for further analysis of (i) nonrotating and (ii) rotating cells. Images are median filtered over the 300‐frame TIRF recordings. (B) Fluorescence intensity line profiles (dotted line in A(i)) from epifluorescence images of surface‐adhered cells expressing variously tagged MotB constructs. (C) Quantification of motor‐associated fluorescence signals from TIRF images of rotating tethered cells (A(ii)). Motor fluorescence is reported as signal‐to‐noise ratio (SNR, maximum fluorescence divided by background, SNR analyzed data are presented in Supporting Information Table 3). Each point represents an individual motor of a single cell; mean ± SD is shown for *n* number of motors or cells. *n* = 5 for wild‐type (WT, i.e., without GFP/iLOV tagged in the MotB), 7 for N‐term‐GFP, 5 for N‐term‐iLOV, 13 for v2‐GFP, 12 for v2‐iLOV, 7 for N‐term‐GS‐GFP, 10 for N‐term‐GS‐iLOV, 10 for v2‐GS‐GFP, and 9 for v2‐GS‐iLOV. Here, “GS” indicates the linker with two amino acids of glycine–serine. N‐terminal GFP fusion (N‐term‐GFP), both with and without GS linkers, showed significantly increased fluorescence compared with other constructs (****p < 0.0001; **p < 0.005 unpaired *t* test). GFP, green fluorescent protein; iLOV, improved light‐oxygen‐voltage.

We measured the fluorescence for both GFP and iLOV‐tagged MotB in two ways. First, by measuring the fluorescence intensity of nonrotating surface‐adhered cells across a region of interest, and second, by measuring the fluorescence at the motor of a rotating tethered cell (Figure 4). The first method allows us to quantify the fluorophore intensity and membrane localization, and the second method validates the recruitment of fluorescent stator complexes to motors. The cytoplasmic variant, GFP tagged to the N‐terminal of MotB (N‐term‐GFP), showed fluorescence as a halo along the edge of the cell, indicating membrane localization. The fluorescence intensity of N‐term‐iLOV was approximately twofold lower than the intensity of N‐term‐GFP (Figure 4B). For all the periplasmic variants (v2), no fluorescent halo was observed along the edge of the cell, for either iLOV or GFP‐tagged MotB, and the fluorescence intensity was equivalent to the autofluorescence of the nonfluorescent background strain (Figure 4B). Insertion of short flexible GS linkers did not change the fluorescence intensity inside the regions of interest shown (Figure 4B and Supporting Information Figure 9). As a positive control, we used monomeric mCherry, which has shown brighter fluorescence in the bacterial periplasm than GFP (Pena et al. 2020). As with GFP and iLOV‐tagged periplasmic variants (v2), mCherry‐tagged MotB constructs were motile but showed limited fluorescence (Supporting Information Figure 10).

We further quantified the fluorescence of active motor by measuring fluorescence over rotating tethered cells (Figure 4A(ii)). By median‐filtering the 300 frames of cell‐rotating video, we obtained a single bright spot (i.e., the motor attachment point around which the cell body was rotating) and quantified the fluorescence of this spot to infer the relative number of stators associated with the motor (Figure 4A,C) (Lele et al. 2013). Similarly to the nonrotating cell measurements, N‐term‐GFP showed higher fluorescence compared with the other constructs. Insertion of GS linkers did not improve the fluorescence. However, N‐term‐GS‐GFP showed decreased fluorescence compared with N‐term‐GFP upon GS linker insertion.

## 3 Discussion

Fluorescently tagged stators have been useful for quantifying stator dynamics in tethered cells (Leake et al. 2006) as well as in response to various loads applied to the flagellar motor (Tipping et al. 2013). These constructs are useful for measuring precisely when and how many stators are engaged, pushing on the rotor during operating cycles of the flagellar motor. However, these constructs have been previously shown to affect rotational speed and, more significantly, switching frequency (Heo et al. 2017). In this paper, we labeled MotB with the fluorescent proteins GFP and iLOV to engineer new constructs with less impacted stator function than existing constructs.

Previous studies have indicated that the disruption of MotB via insertion or deletion, specifically in/near the plug domain, can decrease cell growth or, in some cases, lead to cell death (Hosking et al. 2006). Our constructs with similar insertions (AsLov2) near the plug domain showed growth rates like wild‐type bacteria with native stators, implying no adverse impact on plug function. Periplasmic variants (v2), that is, fluorescent protein (GFP and iLOV) inserted in/near the plug domain, were motile and powered flagellar rotation with similar speed compared with the cytoplasmic variants (N‐term), both in tethered cell measurements and bead assays. However, these periplasmic variants (v2) may constrain the conformational movements of the plug and reduce the effective transduction of ion flow into torque. Alternatively, the stator binding and unbinding may be affected through interference with the insertion of fluorescent proteins and conformational rearrangements that need to occur for flagellar motor rotation. Interestingly, iLOV in the periplasmic variant (v2) swam faster than the GFP counterpart. GFP with a high molecular size of ~28 kDa compared with iLOV (~13 kDa) could have impacted the flagellar motor, slowing it down (Heo et al. 2017). This study is the first to test the effect of fluorescent proteins with different molecular sizes on bacterial motility; thus, we hypothesized that the small molecular size of iLOV may have resulted in reduced steric and structural interference affecting stator rotation (Deme et al. 2020; Santiveri et al. 2020). For both cytoplasmic (N‐term) and periplasmic variants (v2), inserting short and flexible GS linkers into the iLOV‐tagged MotB improved motility as measured by the spread on semisolid agar plates, but not significantly in tethered cell and bead measurements. Along with bacterial swimming speed, spreading of cell populations in the swim plates depends on growth rate and chemotactic ability (Ha et al. 2014), whereas single‐cell measurements via bead or tethered cell assay directly measure torque and speed output of individual motors. We observed no difference in the growth rate due to the presence of linkers in the strains with the LOV domain, like, iLOV, in the stator (Supporting Information Figure 1). Similarly, in single‐cell or individual motor measurements, we assessed switching properties via bead assays and observed no effect from the addition of linkers (Supporting Information Figures 6–8). Each trace represented the rotation speed of an individual motor over time (Supporting Information Figure 7). Discrete speed fluctuations indicated stator unit exchange events, where changes in torque reflected variations in the number of engaged stators (Reid et al. 2006). These dynamic fluctuations demonstrated that motor output is directly correlated with stator occupancy, providing a real‐time, quantitative readout of stator expression and function.

When MotB is tagged with a fluorescent protein, it would be expected to localize in the bacterial membrane of nonrotating adhered cells and fluoresce as a halo along the edges (Heo et al. 2017; Leake et al. 2006; Tipping et al. 2013). Whereas, in rotating cells, fluorescent MotB would be expected to show distinct foci corresponding to motor localization (Lele et al. 2013). GFP‐tagged MotB in the cytoplasmic variant (N‐term) displayed distinct membrane localization compared with the iLOV counterpart. Likewise, rotating N‐term‐GFP showed brighter puncta at the point of attachment than iLOV. This is likely due to the weak fluorescence of iLOV, that is, low quantum yield and brightness compared with GFP (Mukherjee et al. 2013). This was verified by the low intensity of iLOV (without MotB fusion) when expressed in the bacterial cytoplasm (Supporting Information Figure 11). For the periplasmic variant (v2), no distinct membrane‐localized fluorescence was observable either for iLOV or GFP‐tagged MotB. We speculate that protein folding is disrupted in periplasmic variants (v2). Limited fluorescence in the bacterial periplasm could arise from impeded folding in the periplasm, or maturation defects (Meiresonne et al. 2019), or the oxidizing environment and toxicity due to protein overexpression (Arts et al. 2015; Meiresonne et al. 2017; Wilks and Slonczewski 2007). Monomeric mCherry, which has been observed to fluoresce with greater intensity in the bacterial periplasm than GFP (Pena et al. 2020) also showed limited fluorescence in the periplasmic variants (v2) (Supporting Information Figure 10). Similarly, insertion of GS linkers showed no improvement in the fluorescence of GFP and iLOV for both cytoplasmic (N‐term) and periplasmic variants (v2). On the contrary, these flexible short GS linkers diminished the fluorescence intensity in N‐term‐GFP constructs. Future works such as using iLOVs with improved brightness (Liang et al. 2022), rigid linkers of varying lengths (Heo et al. 2017) and targeting new sites between the plug and PG binding domain of the MotB stator protein could generate improved labeled stators for experimental use.

## 4 Conclusion

This paper tested the effect of tagging MotB near the plug domain with fluorescent proteins. Our findings showed that insertion of GFP and iLOV near the plug domain did not disrupt the function of the stator complex, but also did not fluoresce brightly, and these constructs were not suitable for single‐molecule measurements of stator binding and unbinding. We have successfully shown that periplasmic sites near the plug are suitable for the addition of either fluorescent reporters or light‐switchable domains, but further work is needed to improve both the fluorescence and light‐response of these domains to enable direct light‐activation of the flagellar stator complex.

## 5 Materials and Methods

### 5.1 Plasmid Construction

Four different chimeric constructs of *E. coli* MotB and GFP or LOV protein domains were designed in silico and synthesized as gBlocks (IDT) for experimental testing in vivo in *E. coli*. All constructs employed in the study were cloned into the pBAD33 vector (chloramphenicol resistant) and expressed using an Arabinose‐inducible pBAD expression system (Guzman et al. 1995). To construct plasmids for four variants (v1, v2, v3, and v4), fragments containing AsLOV2 and MotB with two restriction sites were PCR amplified from DNA fragments, then double‐digested (SalI and PstI from New England Biosciences [NEB]), ligated (T4 DNA‐Ligase, NEB), and transformed into the background strains. Two background strains were used: RP3087 (∆motB, for swim plate assay) (Blair et al. 1991) and SYC35 (∆motA ∆motB fliC‐sticky) for transformation (Islam et al. 2021). Similarly, restriction‐digestion was used to construct plasmids containing iLOV, GFP, and mCherry in the N‐terminal and v2 positions of MotB. Quickchange site‐directed mutagenesis was employed to insert GS linkers in these plasmids at the N‐ and C‐terminal flanks of the chimeric insert. The sequences of primers used for restriction‐ligation and site‐directed mutagenesis are listed in Supporting Information Table 4.

### 5.2 Bacterial Sample Preparation

Frozen aliquots of cells (grown to saturation and stored in 25% glycerol at −75°C) were aerobically grown overnight in 5 mL of Lysogeny Broth (LB, 10 g L^−1^ bacto‐tryptone, 5 g L^−1^ yeast extract, and 10 g L^−1^ NaCl) at 37°C in a continuous shaking incubator at 200 rpm. The next day, a new culture was started by diluting 50 µL of the overnight culture in 5 mL of Tryptone Broth (TB, 10 g L^−1^ bacto‐tryptone, 5 g L^−1^ NaCl) containing 1.33 mM l‐arabinose at 30°C. Cells were grown for about 6 h, shaking at 200 rpm, to a final optical density at 600 nm of 0.5–0.6. All the culture media contained 100 µg mL^−1^ ampicillin and 25 µg mL^−1^ chloramphenicol.

### 5.3 Swim Plate Assay

For swim plates, a single colony of bacteria was inoculated in a semisolid agar medium (containing 0.3% agar) and incubated at 30°C. To test the effect of blue light in bacteria containing light‐responsive domains, such as LOV, we built a blue light projector system to illuminate swim plates from the top (Zhang et al. 2020). To build this system, a blue light‐emitting diode (LED) of 465 nm (Meccanixity) was installed in the car door light projector (Supporting Information Figure 2). The LED was powered by a 0–5‐V direct current power source. The swim plate inoculated with bacteria was placed 12 cm below the blue LED, and the entire system was placed inside the incubator at 30°C. Swim plates were illuminated with 150 lx for 24 h at 30°C. The swim ring was imaged using a gel doc imager (Bio‐Rad) and measured using ImageJ software.

### 5.4 Tethered Cell Assay

The rotational speed of cells tethered to the microscope glass slide via a single flagellum was measured to analyze individual flagellar motors. Bacterial cells with short sticky filaments (SYC35, i.e., *∆motA* ∆*motB fliC*‐sticky) where MotA and MotB stator proteins were coexpressed on two different plasmids (Supporting Information Table 2) were tethered to a glass surface. An inverted phase contrast microscope (Nikon) with a 20X objective lens was used to image the rotation of the tethered cells. The 20‐s‐long videos were acquired with a camera acquisition rate of 75 fps and the rotational speed was measured using LabView software. The number of cells used for the measurements and analysis is listed in the Supporting Information Table 2.

### 5.5 Bead Assay

The same bacterial strains were used for both the bead assay and the tethered cell assay. To obtain short flagellar stubs, bacteria were mechanically sheared by passing 1 mL of cell culture back and forth between two syringes connected by 21‐gauge needles and a thin tube (Gabel and Berg 2003). Cells were then centrifuged (3000 rpm for 3 min) and resuspended in a motility buffer (MB: 10 mM potassium phosphate, 0.1 mM ethylenediaminetetraacetic acid, 10 mM lactic acid, pH 7.0). Experiments were performed in a “tunnel slide” made of two coverslips (Menzel‐Glaser #1.5) separated by a layer of parafilm, with a tunnel cut into it, melted to the coverslips. Two laser‐cut holes in the top coverslip allowed fluid exchange. The slides were cleaned with ethanol and sterile water before use. First, 100 µL of poly‐l‐lysine (Sigma‐Aldrich P4707) was introduced into the tunnel slide and incubated for 2 min, followed by flushing with 200 µL of MB. Next, 100 µL of cell suspension was flushed through the slide, allowing cells to settle on the coverslip for 10 min. Unattached cells were washed out with MB. Finally, a 1/300 dilution of polystyrene beads (1.1 µm in diameter, Sigma‐Aldrich) in MB was flushed through. Beads were allowed to spontaneously bind to “sticky” truncated filaments and after about 10 min unattached beads were washed out with MB. Motor rotation was measured by monitoring the rotation of the bead using a custom‐made bright‐field microscope. The sample was illuminated with a 660 nm LED (Thor labs, M660L3) and imaged with a 100 × 1.45‐NA objective (Nikon) onto a high‐speed CMOS camera (Optronics CL600x2/M). Acquisitions of rotating beads were taken at a sampling rate of 1 kHz.

### 5.6 Differential Dynamic Microscopy

A single colony was chosen from LB agar single colony streak plates and inoculated in liquid LB broth for overnight culture at 37°C, at 180 rpm in a shaking incubator. From this, 350 µL of overnight culture was added to 35 mL of TB broth and incubated at 30°C in a shaking incubator for 4–5 h till the OD600 reached 0.5–0.6. This was then diluted by five times by adding 5 mL of PBS to 1 mL of subculture. Then, 50–70 µL of the diluted sample (OD600 around 0.1–0.2) was loaded in a rectangular capillary tube (VitroTubes), sealed with vaseline on both ends of the capillary, and imaged with an Olympus IX‐71 inverted microscope with ×10 phase contrast and Köhler illumination. Samples were imaged in the middle plane of the capillary tube. Videos of 1 min at 100 fps (i.e., 6000 frames) were acquired at a frame size of 512 × 512 pixels using a Mikrotron MC‐1362 camera connected to a frame grabber card (Euresys Grablink). LabView code was used to process the video, followed by using in‐house MATLAB codes for the analysis and data visualization (Wilson et al. 2011). The mean swim velocity for each bacterial strain was obtained by fitting the DDM parameters versus *q* values, which is explained in detail in the review paper (Al‐Shahrani and Bryant 2022).

### 5.7 Fluorescence Assay

An Elyra‐7 (Zeiss) microscope was used to test the fluorescence of bacterial strains expressing MotB tagged with GFP and fluorescent LOV domain (iLOV). An oil immersion objective lens with 63 × NA‐1.46 was used for all the bacterial strains. Laser light (0.24 mW) of 488 nm wavelength was used to excite GFP and iLOV. Bacterial samples were prepared as in the tethered cell assay and bead assay to test fluorescence, along with rotation. In all, 100 image frames were acquired with an exposure time of 100 ms and an image of size 512 × 512 pixels. A single image was generated from a median filter of the 300 images.

### 5.8 Data Analysis

For the bead assay, the data analysis was performed using custom LabView and Python scripts. The *x*(*t*) and *y*(*t*) positions of the rotating bead were determined by using a cross‐correlation analysis of the bead image with a numerically generated kernel pattern (Lipfert et al. 2011). The drift of the circular trajectory was corrected by subtracting a linear interpolation of *x* and *y* from their respective raw values. The elliptical trajectories of the beads, assumed to be the projection of a tilted circle, were transformed into circles by stretching the minor axis of the ellipse. Speed was calculated by taking the derivative of the angle, and the speed traces were filtered using a 70‐ms window median filter. Switching events were detected by identifying crossings of two thresholds: the positive speed threshold was set at 2/3 of the mean positive speed (Bai et al. 2010; Heo et al. 2017), and the negative threshold was set at 2/3 the mean negative speed, except in cases where the mean negative speed was greater than −3 Hz, in which case the negative threshold was set to −3 Hz. The switching frequency of the motor was calculated as the number of detected switching events divided by the duration of the measurement. MATLAB and Prism GraphPad were used to analyze and plot the data. Statistical analysis was performed using an unpaired *t* test (except for the swim plate assay, comparing motility in the dark and light conditions of the same bacterial strain, where a paired *t* test was performed). Data were shown as mean ± standard deviation with error bars. Significance levels while comparing two or more data sets were presented as *p* values.

## Author Contributions

**Jyoti P. Gurung:** writing – original draft, investigation, visualization, writing – review and editing, methodology, data curation, formal analysis. **Pietro Ridone:** investigation, writing – review and editing, methodology, validation. **Anaïs Biquet‐Bisquert:** investigation, methodology, validation, visualization, software, writing – review and editing, formal analysis. **Gary Bryant:** writing – review and editing, methodology. **Francesco Pedaci:** writing – review and editing, methodology, software, formal analysis, funding acquisition, resources, supervision. **Ashley L. Nord:** funding acquisition, writing – review and editing, resources, supervision. **Matthew A. B. Baker:** funding acquisition, writing – original draft, writing – review and editing, conceptualization, methodology, validation, visualization, project administration, supervision, resources.

## Conflicts of Interest

The authors have nothing to report.

## Supporting information


**Supplementary Figure 1:** Line graph of growth curves, recorded as absorbance at 600 nm, incubated at 37°C for 24 hours. **Supplementary Figure 2:** Effect of blue light (465 nm, 150 lux intensity) in the motility of bacteria with LOV inserted MotB. **Supplementary Figure 3:** Image of swim plates after 24 hours (left) and 48 hours (right) of incubation at 30°C. Bacterial strains. **Supplementary Figure 4:** Swim plate assay depicting the motility status of v3 and v4. **Supplementary Figure 5:** Sanger sequencing result of motB gene fragment amplified from the bacterial genome. **Supplementary Figure 6:** Motor speed traces of individual CCW‐biased motors rotating 1.1 μm beads. **Supplementary Figure 7:** Single‐molecule speed traces of bacterial flagellar motors attached with rotating 1.1 μm beads, showing dynamic stator behaviour. **Supplementary Figure 8:** Switching frequency distributions plotted for iLOV and GFP tagged MotB in N‐terminal and v2 position from all the individual speed traces of (n) number of motors (Supplementary Fig. 7). **Supplementary Figure 9:** Microscopic images of GFP and iLOV tagged MotB constructs. **Supplementary Figure 10:** Motility and fluorescence of mCherry‐v2‐GS bacterial strain. **Supplementary Figure 11:** Fluorescence of cytoplasmic expression of iLOV and GFP fluorescent proteins. **Supplementary Table 1:** List of the average swim velocity (mean ± with standard deviation) from differential dynamic microscopy (DDM). **Supplementary Table 2:** List of the average rotational speed of tethered cell (mean ± with standard deviation) from tethered cell assay. **Supplementary Table 3:** List of the average motor fluorescence of a rotating tethered cell (mean ± with standard deviation) i.e., ratio of maximum fluorescence intensity and background intensity (Signal to noise ratio – SNR). **Supplementary Table 4:** List of primers used in this work. GGCAGC: GS linker nucleotide sequence.

## Data Availability

The data that support the findings of this study are available in the supporting material of this article.
