# Supplementary material for: Insertion of Fluorescent Proteins Near the Plug Domain of MotB Generates Functional Stator Complexes
Source: Microbiologyopen. 2025 Sep 15;14(5):e70056. doi: 10.1002/mbo3.70056 (PMC12434310; doi:10.1002/mbo3.70056)
Supplement: Supplementary file 1 — Supplementary Figure 1: Line graph of growth curves, recorded as absorbance at 600 nm, incubated at 37°C for 24 hours. Supplementary Figure 2: Effect of blue light (465 nm, 150 lux intensity) in the motility of bacteria with LOV inserted MotB. Supplementary Figure 3: Image of swim plates after 24 hours (left) and 48 hours (right) of incubation at 30°C. Bacterial strains. Supplementary Figure 4: Swim plate assay depicting the motility status of v3 and v4. Supplementary Figure 5: Sanger sequencing result of motB gene fragment amplified from the bacterial genome. Supplementary Figure 6: Motor speed traces of individual CCW‐biased motors rotating 1.1 μm beads. Supplementary Figure 7: Single‐molecule speed traces of bacterial flagellar motors attached with rotating 1.1 μm beads, showing dynamic stator behaviour. Supplementary Figure 8: Switching frequency distributions plotted for iLOV and GFP tagged MotB in N‐terminal and v2 position from all the individual speed traces of (n) number of motors (Supplementary Fig. 7). Supplementary Figure 9: Microscopic images of GFP and iLOV tagged MotB constructs. Supplementary Figure 10: Motility and fluorescence of mCherry‐v2‐GS bacterial strain. Supplementary Figure 11: Fluorescence of cytoplasmic expression of iLOV and GFP fluorescent proteins. Supplementary Table 1: List of the average swim velocity (mean ± with standard deviation) from differential dynamic microscopy (DDM). Supplementary Table 2: List of the average rotational speed of tethered cell (mean ± with standard deviation) from tethered cell assay. Supplementary Table 3: List of the average motor fluorescence of a rotating tethered cell (mean ± with standard deviation) i.e., ratio of maximum fluorescence intensity and background intensity (Signal to noise ratio – SNR). Supplementary Table 4: List of primers used in this work. GGCAGC: GS linker nucleotide sequence. [file MBO3-14-e70056-s001.pdf]

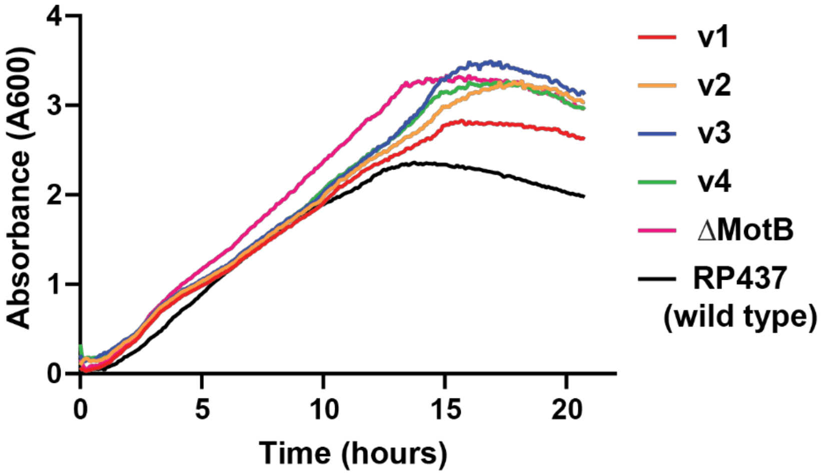

Supplementary Fig. 1. Line graph of growth curves, recorded as absorbance at 600 nm, incubated at 37°C for 24 hours. Bacterial strains: MotB tagged with AsLOV2 (v1, v2, v3, v4), MotB deleted strain as background strain (ΔMotB), and wild-type motile strain as positive control (RP437). Absorbance was recorded every 15 minutes at time intervals for 24 hours. Average absorbance was plotted versus time, taken from four replicates for each bacterial strain.

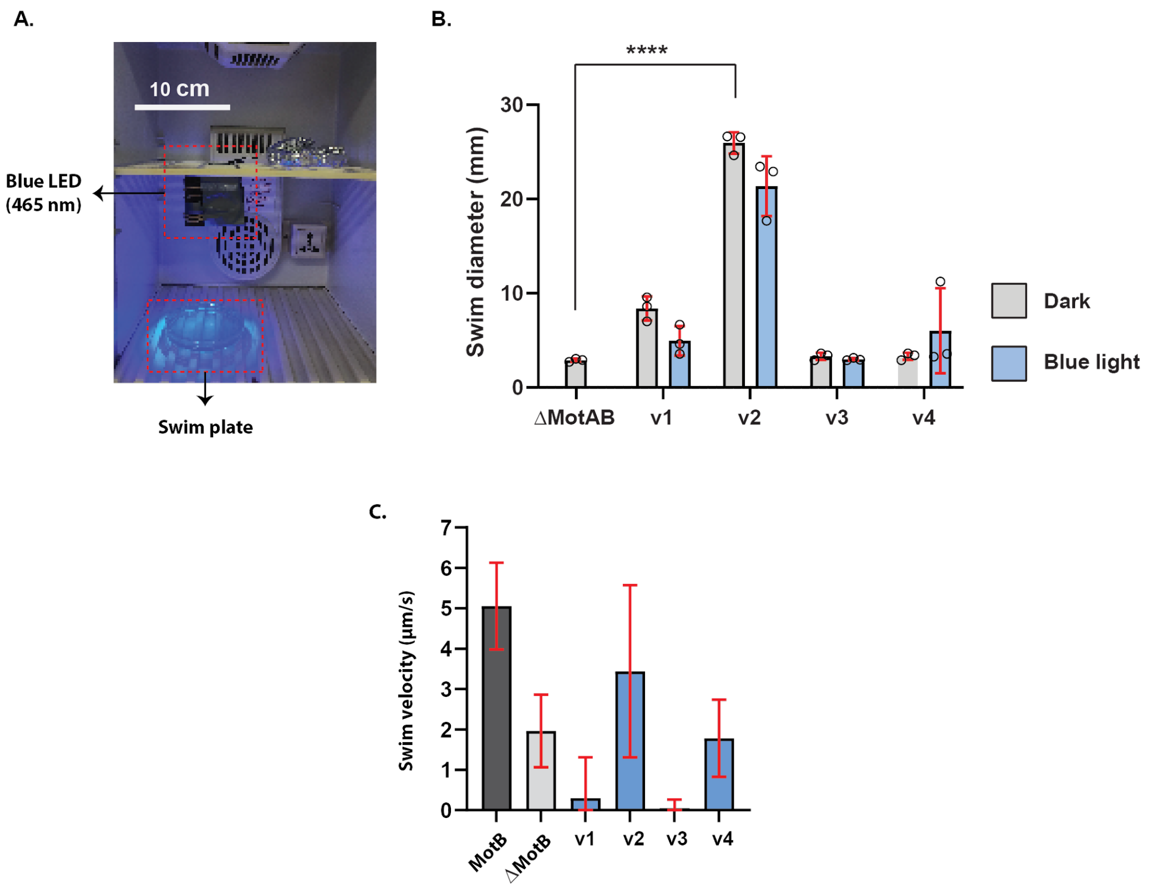

Supplementary Fig. 2. Effect of blue light (465 nm, 150 lux intensity) in the motility of bacteria with LOV inserted MotB. Bacterial strains; MotB – wildtype motile (positive control), Δ*motAB* and Δ*motB* - non-motile (negative control), LOV domain inserted MotB; v1 (before plug domain), v2 (after plug domain), v3 (before PG domain), and v4 (after PG domain). Blue-coloured bar – blue light illumination and light-grey coloured – dark condition. (A) Blue light illumination system (scale bar of 10 cm). The swim plate was placed under the blue LED projector (465 nm) at a distance of ~ 12 cm inside an incubator. The swim plate and blue LED are labelled and denoted by a dotted red rectangle in the figure above. The intensity of blue light was measured at ~150 lux by lux meter. (B) Bar graph of swim ring diameter (mean ± standard deviation, triplicates data) measured from swim plate assay. (C) Swimming velocity of bacterial strains in liquid media measured by differential dynamic microscopy (DDM).

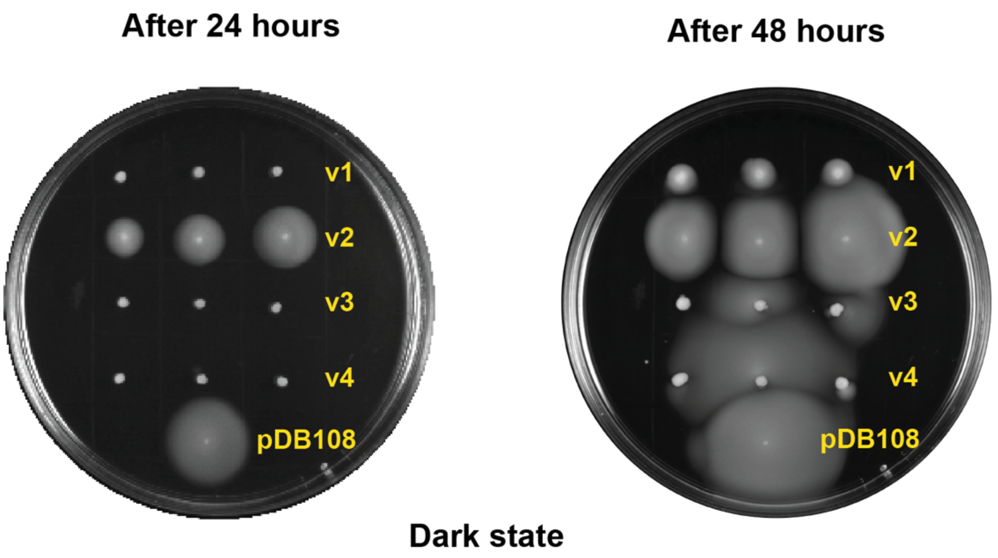

Supplementary Fig. 3. Image of swim plates after 24 hours (left) and 48 hours (right) of incubation at 30°C. Bacterial strains: MotB tagged with AsLOV2 (v1, v2, v3, v4), and wild-type motile strain as a positive control (pDB108 – $MotA_5MotB_2$ expressing bacterial strain). Three colonies for each bacterial strain were inoculated in a swim plate (except for pDB108 i.e., equivalent to wildtype motile strain expressing MotAB stator proteins).

.

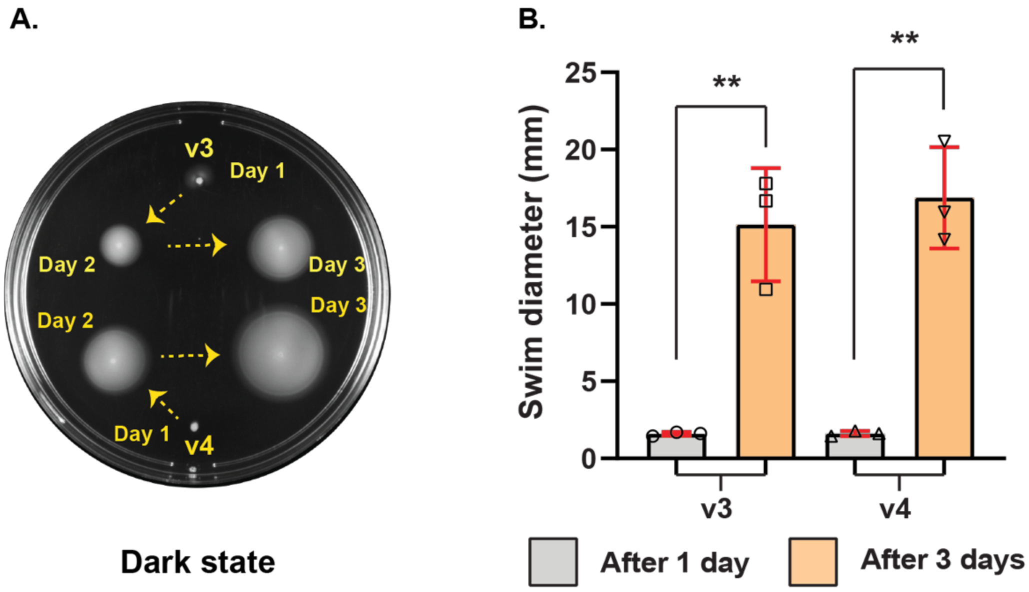

Supplementary Fig. 4. Swim plate assay depicting the motility status of v3 and v4. (A). Image of swim plate showing the motility of colonies after incubating for 1 day, 2 days, and 3 days at 30°C. (B). Bar graph of swim diameter (mean ± standard deviation) of v3 and v4 measured for colonies incubated for 1 day and 3 days at 30°C.

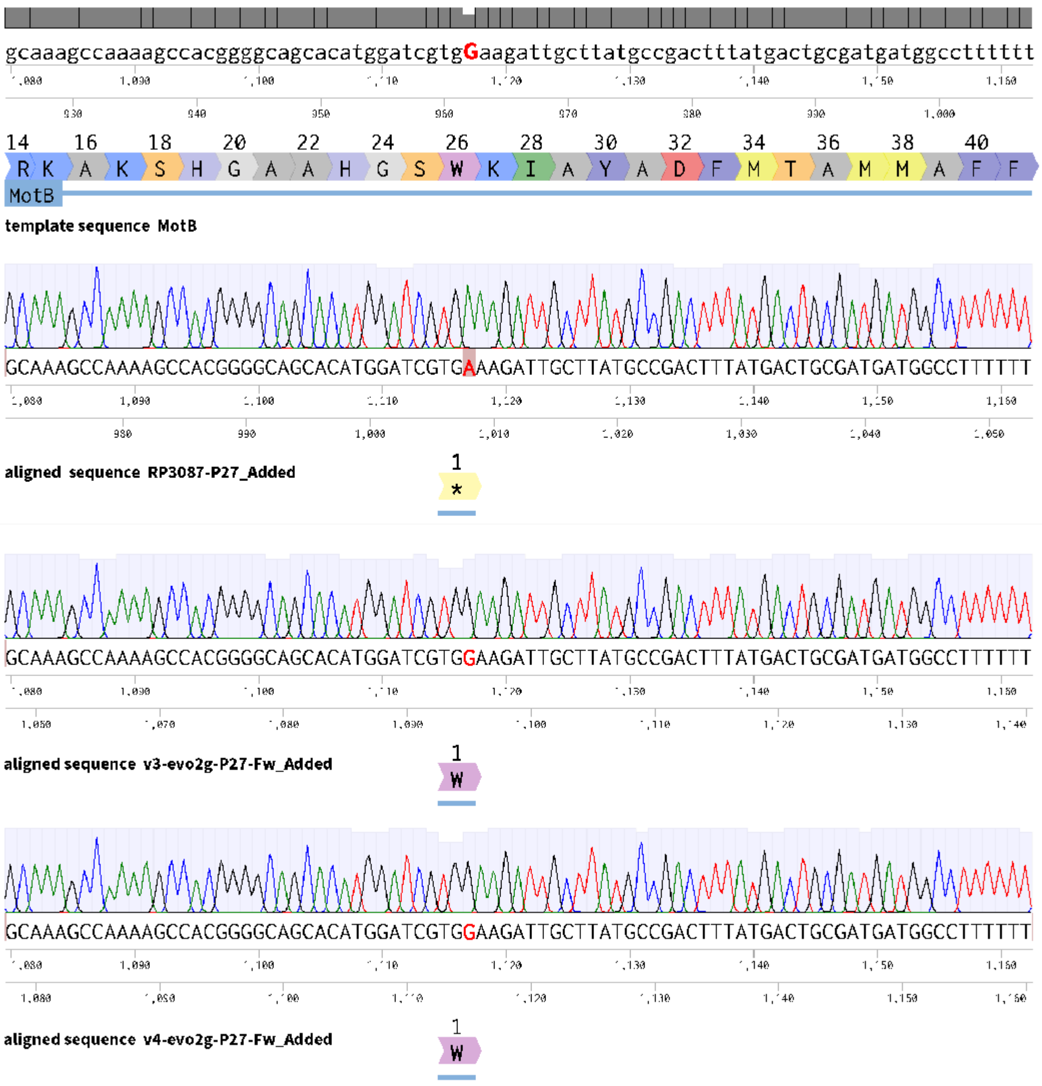

Supplementary Fig. 5. Sanger sequencing result of motB gene fragment amplified from the bacterial genome. In RP3087 i.e., ΔMotB background bacterial strain, 26th amino acid tryptophan (**W**) was converted to stop codon (*****) by single nucleotide mutation (TG**G** to TG**A**) to stop the translation of MotB. After 2-3 days of incubation in a swim plate at 30°C, variants v3 and v4 (contains plasmid expressing LOV domain inserted at PG binding domain of MotB in ΔMotB background strain) rescued its motility by reverting this mutation from TG**A** to TG**G** (i.e., from stop codon '*****' to tryptophan '**W**').

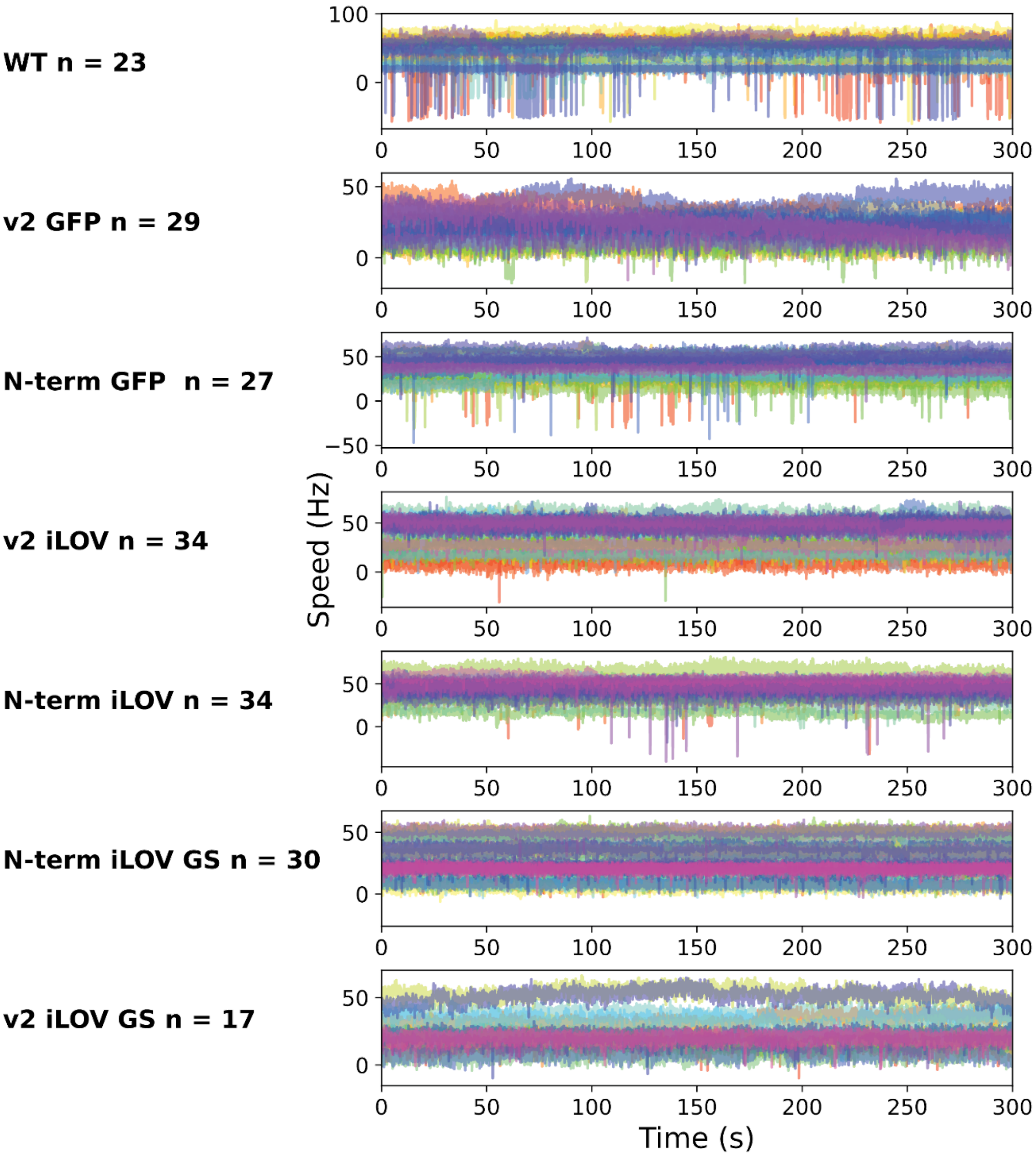

Supplementary Fig. 6. Motor speed traces of individual CCW-biased motors rotating 1.1 µm beads. Positive speeds indicate counterclockwise (CCW) rotation, while negative speeds indicate clockwise (CW) rotation. The number of motors measured is 23 for WT, 29 for v2 GFP, 27 for N-term GFP, 34 for v2-iLOV and 34 for N-term iLOV.

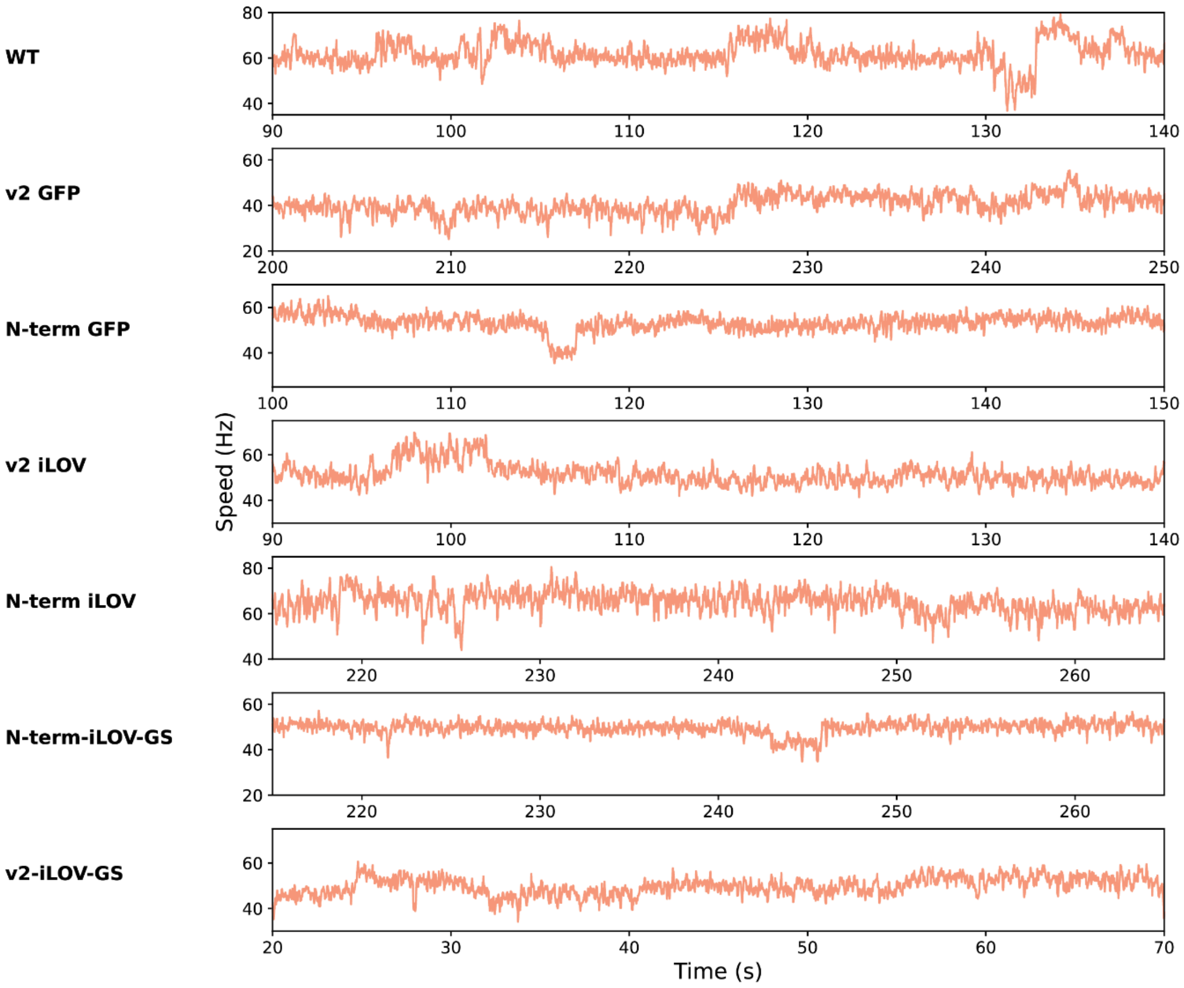

Supplementary Fig. 7. Single-molecule speed traces of bacterial flagellar motors attached with rotating 1.1 µm beads, showing dynamic stator behaviour. Each trace (orange-colored) represents the rotational speed of an individual motor over time. The observed fluctuations correspond to the stator units dynamically associating and dissociating from the motor. This inset of single motor measurements (from Supplementary Fig. 6) was prepared to show the expression of stator units in single-molecule resolution for iLOV/GFP tagged MotB constructs.

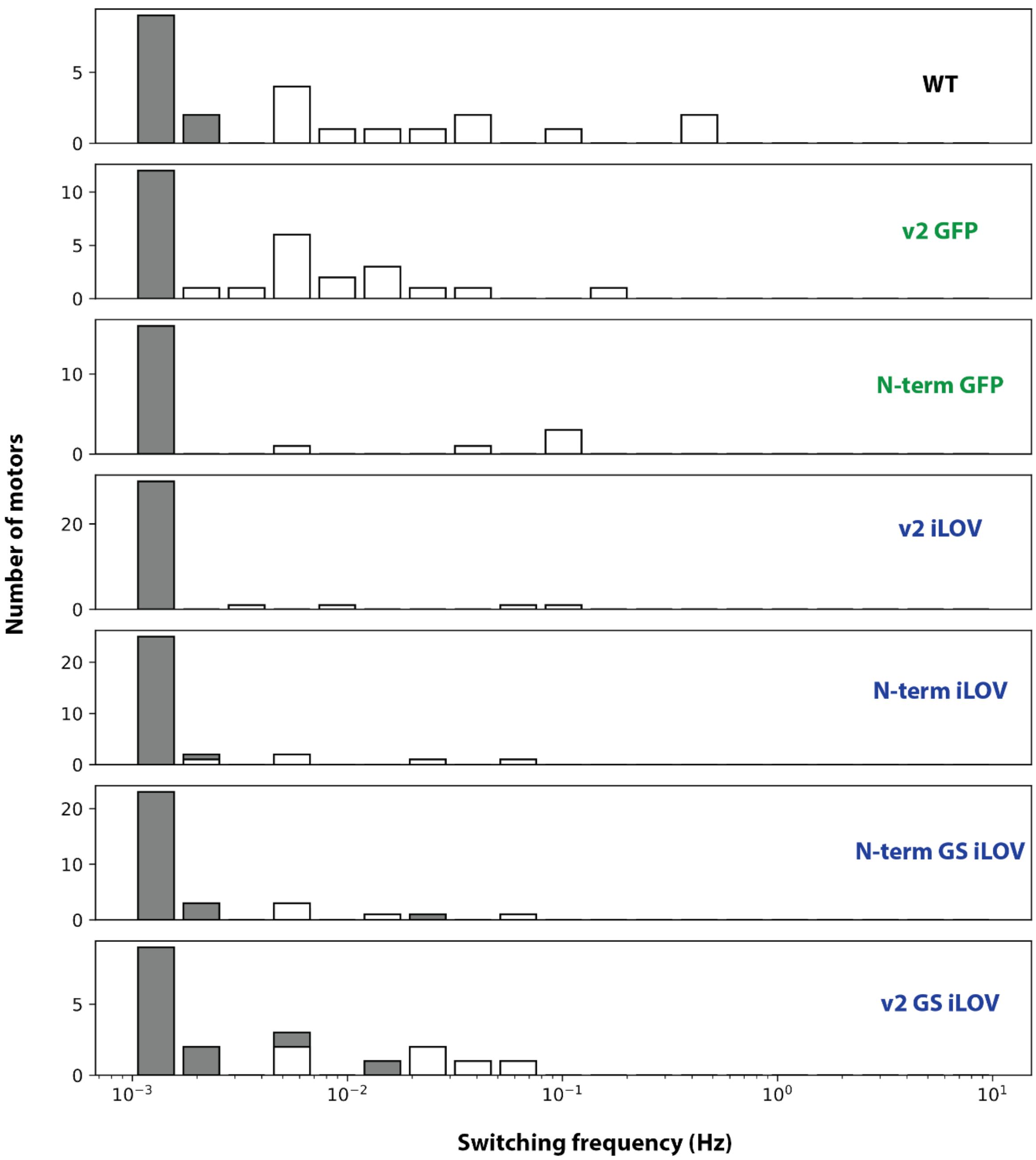

Supplementary Fig. 8. Switching frequency distributions plotted for iLOV and GFP tagged MotB in N-terminal and v2 position from all the individual speed traces of (n) number of motors (Supplementary Fig. 7). Grey bar denotes the motors that did not switch during the measurements. The number of motors (n) measured was 23 for WT, 29 for v2 GFP, 27 for N-terminal GFP, 34 for v2-iLOV, 34 for N-terminal iLOV, 30 for N-terminal iLOV GS, and 17 for v2 iLOV GS. Here, 'GS' indicates the linker with two amino acids of Glycine-Serine.

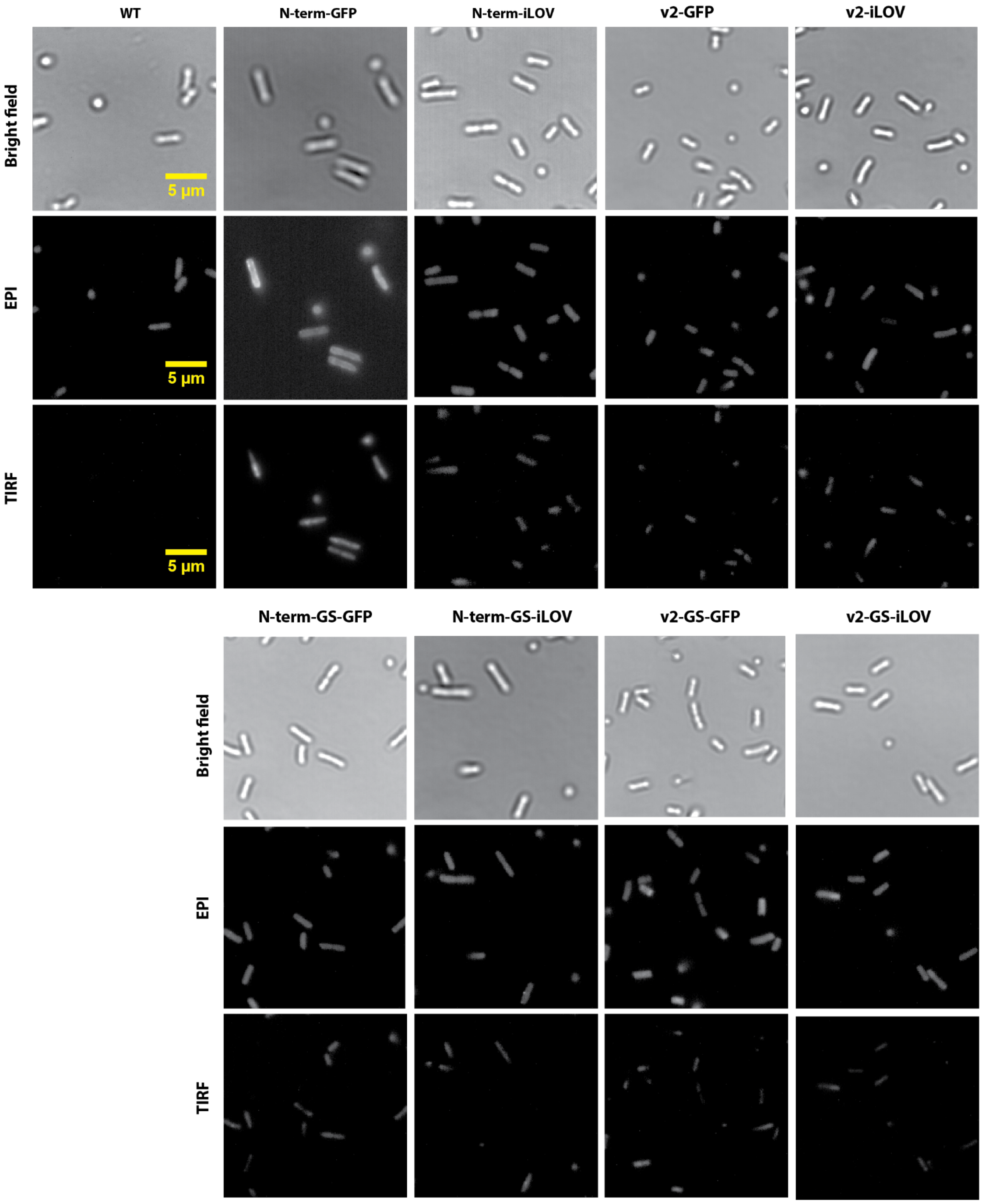

Supplementary Fig. 9. Microscopic images of GFP and iLOV tagged MotB constructs. It included the same field of view of bright field, EPI, and TIRF images for each construct. Median filtered images of 300-frame bright field, EPI, and TIRF fluorescence recordings. Bacterial samples were excited by a 488 nm laser with power 0.24 mW and exposure time of 100 ms.

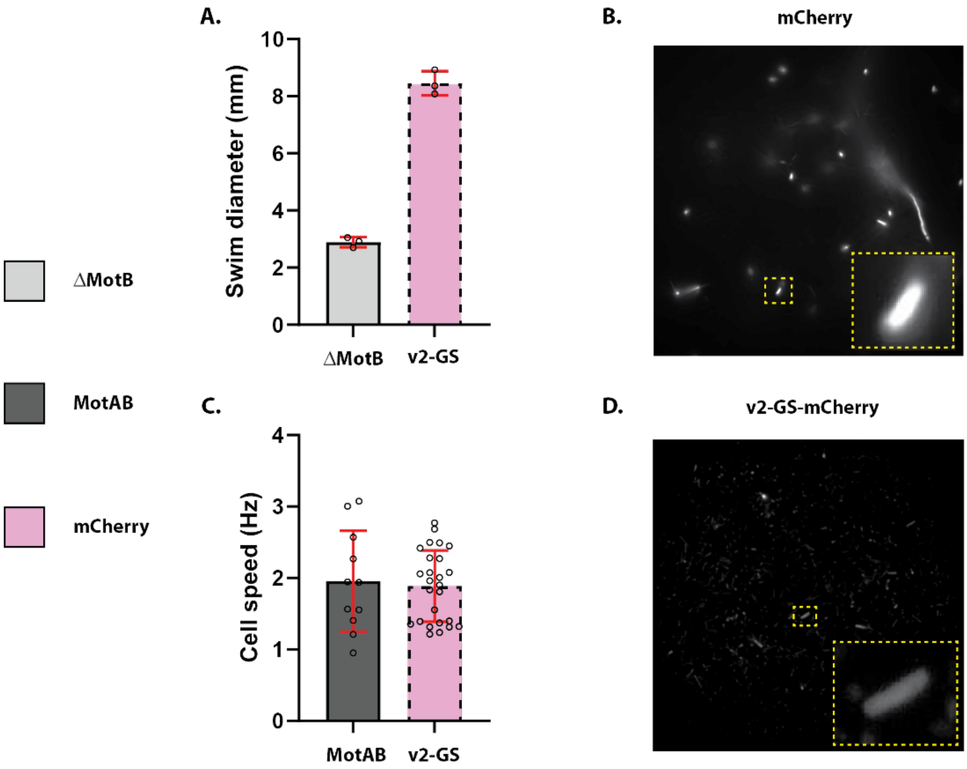

Supplementary Fig. 10. Motility and fluorescence of mCherry-v2-GS bacterial strain. (A) Bar graph of swim ring diameter (mean ± standard deviation, triplicates data) measured for the bacterial strains tested (B) Bar graph of rotational speed (mean ± standard deviation) for the bacterial strains tested. (C) Cytoplasmic expression of mCherry (positive control) (D) MotA + MotB-GFP-v2-GS. 100 frames of fluorescence images were recorded in EPI mode of illumination, excited by a 488 nm laser of 5 mW power and 100 ms of exposure time. All intensity measurements were captured by an sCMOS camera.

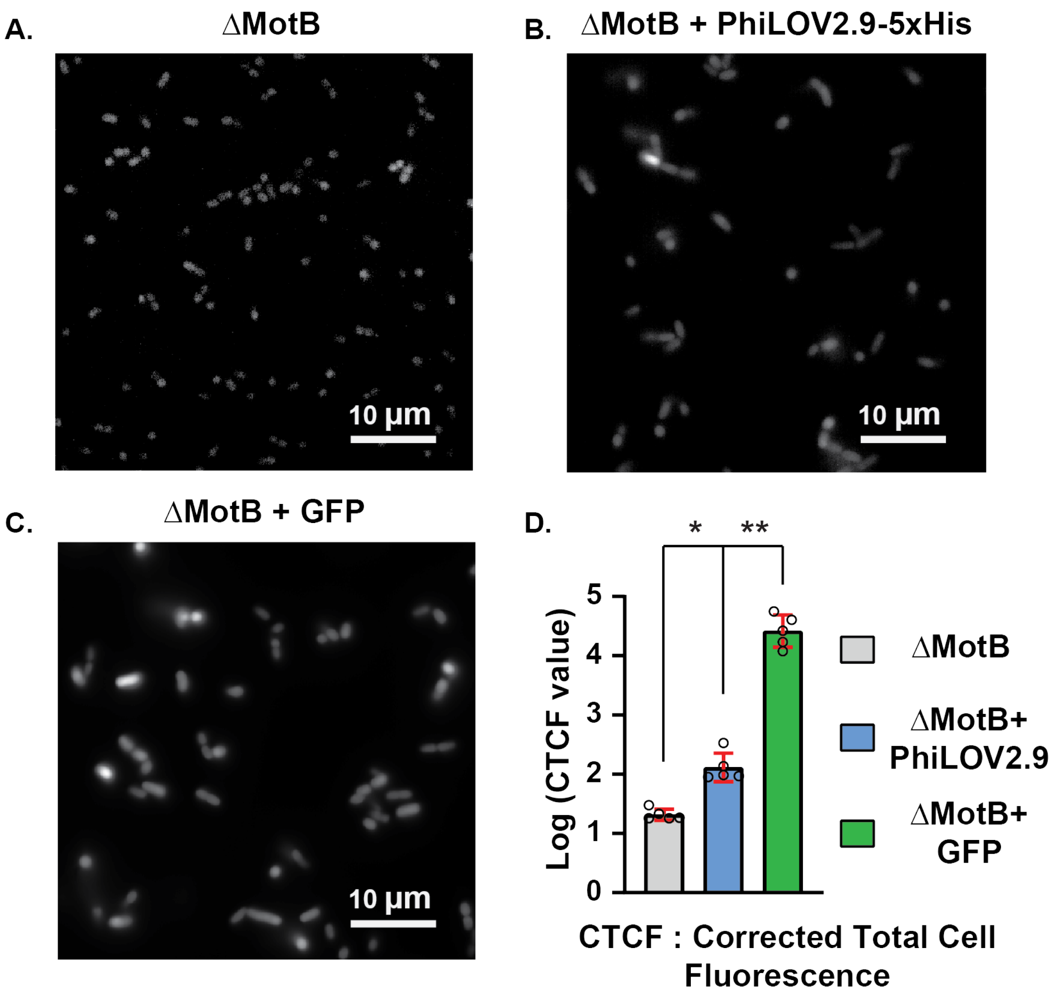

Supplementary Fig. 11. Fluorescence of cytoplasmic expression of iLOV and GFP fluorescent proteins. The minimum pixel intensity was set at 100 for all three images. Fluorescence images (median of 100 frames) of (A) ΔMotB (maximum - 145), (B) ΔMotB + iLOV-5xHis (maximum – 342), and (C) ΔMotB + GFP (maximum - 29357). (D) Fluorescence measurements using corrected total cell fluorescence (CTCF) value. To quantify the fluorescence signal in bacterial cytoplasm, the corrected total cell fluorescence (CTCF) value was calculated. First, a region of interest (ROI) was drawn around whole bacterial cell, then, values for 1) I_D, the integrated density (the sum of pixels in the region of interest), 2) A_{ROI}, the area of the ROI, and 3) F_B, the mean fluorescence of background pixels were measured (as determined by dark pixels outside the boundary of the cell). Finally, CTCF was calculated in ImageJ by using the formula: CTCF = I_D – (A_{ROI} x F_B). Five cells for each bacterial strain were used to calculate the average CTCF value and plotted as a logarithmic value. All intensity measurements were captured by an sCMOS camera.

Supplementary Table 1. List of the average swim velocity (mean ± with standard deviation) from differential dynamic microscopy (DDM).

E. coli strain	Condition	Plasmids	Speed velocity (µm/s)
RP3087	Dark	pMotB	5.1 ± 1.1
RP3087	Dark	--	2.0 ± 0.1
RP3087	Dark	pMotB-LOV-v1	0.3 ± 1.0
RP3087	Dark	pMotB-LOV-v2	3.4 ± 2.1
RP3087	Dark	pMotB-LOV-v3	0.1 ± 0.2
RP3087	Dark	pMotB-LOV-v4	1.8 ± 1.0

Supplementary Table 2. List of the average rotational speed of tethered cell (mean ± with standard deviation) from tethered cell assay.

E. coli strain	Plasmids	No. of cells (n)	Rotational speed (Hz)
SYC35	pMotA, pMotB	15	6.0 ± 3.1
SYC35	pMotA, pGFP-MotB	16	4.1 ± 2.4
SYC35	pMotA, pGFP-MotB-GS	9	3.9 ± 1.9
SYC35	pMotA, pMotB-GFP-v2	16	2.8 ± 1.6
SYC35	pMotA, pMotB-GFP-v2-GS	17	3.5 ± 2.2
SYC35	pMotA, piLOV-MotB	6	5.1 ± 2.2
SYC35	pMotA, piLOV-MotB-GS	17	4.6 ± 2.5
SYC35	pMotA, pMotB-iLOV-v2	19	5.2 ± 2.0
SYC35	pMotA, pMotB-iLOV-v2-GS	14	4.0 ± 2.6

Supplementary Table 3. List of the average motor fluorescence of a rotating tethered cell (mean ± with standard deviation) i.e., ratio of maximum fluorescence intensity and background intensity (Signal to noise ratio – SNR).

***E. coli* strain**	**Plasmids**	**No. of motor/cells (n)**	**Motor fluorescence (SNR)**
SYC35	pMotA, pMotB	5	1.22 ± 0.04
SYC35	pMotA, pGFP-MotB	7	3.0 ± 0.46
SYC35	pMotA, pGFP-MotB-GS	7	1.50 ± 0.17
SYC35	pMotA, pMotB-GFP-v2	13	1.20 ± 0.05
SYC35	pMotA, pMotB-GFP-v2-GS	10	1.18 ± 0.04
SYC35	pMotA, piLOV-MotB	5	1.21 ± 0.07
SYC35	pMotA, piLOV-MotB-GS	10	1.35 ± 0.13
SYC35	pMotA, pMotB-iLOV-v2	12	1.21 ± 0.08
SYC35	pMotA, pMotB-iLOV-v2-GS	9	1.14 ± 0.08

Supplementary Table 4. List of primers used in this work. *GGCAGC*: GS linker nucleotide sequence

**Primers**	**Sequence**
**Restriction-Digestion**	
P20	CAGTGAATGGGGGTAAAT
P24	GGTTGGACTCAAGACGATAG
**Site-directed mutagenesis**	
GFP-MotB link Fw	GGCAGCATGAAAAATCAGGCTCACC
GFP-MotB link Rv	CTTGTACAGTTCGTCCATG
iLOV-MotB link Fw	GGCAGCATGAAGAATCAAGCGCATC
iLOV-MotB link Rv	TTTATCATCATCATCTTTATAATCGCT
v2-EGFP-909a	GGATGAGCTTTACAAGGGCAGCCCCCTTGCTACCGC
v2-EGFP-192b	TCCCCTTTACTCACCATGCTGCCCGTACGGAAGTATTCGG
